# Challenges of primary healthcare nurses regarding the integration of HIV and AIDS services into primary healthcare in Vhembe district of Limpopo province, South Africa

**DOI:** 10.4102/curationis.v42i1.1849

**Published:** 2019-01-28

**Authors:** Azwidihwi R. Tshililo, Lizzy Mangena-Netshikweta, Livhuwani H. Nemathaga, Mary Maluleke

**Affiliations:** 1 Department of Advanced Nursing Science, University of Venda, South Africa

## Abstract

**Background:**

Integration of human immunodeficiency virus and acquired immune deficiency syndrome (HIV and AIDS) services into primary healthcare (PHC) is a key public health approach to achieving universal access to antiretroviral therapy (ART). Despite the government’s efforts of integrating HIV services into PHC, an insufficient number of PHC staff and inadequate infrastructure are challenging when integrating HIV and AIDS services into PHC. This study explored the challenges of PHC nurses regarding the integration of HIV and AIDS services into PHC.

**Objectives:**

The aim of the study was to explore the challenges of PHC nurses regarding the integration of HIV and AIDS services into PHC.

**Method:**

An exploratory, descriptive and contextual qualitative research design utilising face-to-face semi-structured interviews was conducted with 12 PHC nurses from selected clinics and health centres in the Vhembe district of Limpopo province.

**Results:**

Two main themes emerged from data analysis which included challenges related to healthcare recipients and challenges related to healthcare providers.

**Conclusion:**

Clear policies on the integration of HIV and AIDS services into PHC should be available and should include strategies to promote HIV testing and counselling, adherence to ART and scheduled appointments, disclosure of HIV status as well as revising the human resource policy to reduce workload.

## Introduction

Integrating HIV services into primary healthcare (PHC) was adopted by the government of South Africa as a key to achieving universal access to antiretroviral therapy (ART). The integration of HIV services into primary healthcare has the benefit of promoting coherent and holistic services. It increases access to care, allows more people to be treated, improves retention in care, saves costs and potentially leads to improved patient outcomes (Crowley & Stellenberg 2014). Despite the government’s efforts of integrating HIV services into PHC, an insufficient number of PHC staff and inadequate infrastructure is problematic when such integration is taking place. Therefore, the study explored the challenges of PHC nurses regarding the integration of HIV/AIDS services into PHC in Vhembe district of Limpopo province, South Africa.

### Literature review

Integration of HIV and AIDS services into PHC is a sharing of services and resources for HIV care with existing PHC programmes. These include clinic space, clinicians, health education, pharmacy, laboratory services and training. People living with HIV (PLWHIV) are sharing services and resources with other patients at PHC clinics and health centres (Church et al. 2015). The study by Topp et al. (2013) concluded that integration of HIV care and treatment is increasingly being supported as a way to achieve the three-way aims of improved clinical care, better public health and health system outcomes and more cost-efficient programming. Furthermore, the study by Odeny et al. (2013) revealed that the integration of HIV and AIDS services into PHC allows people to access the healthcare they require regardless of HIV status. Integrating HIV and AIDS services into PHC further enhances and improves patient care and yield improvements in access to care, quality of care and efficiency in service delivery. Service integration also improves care and reduces missed opportunities for key interventions such as HIV testing, provision of ART, prevention of mother to child transmission (PMTCT), and adherence support. Additionally, service integration provides more comprehensive care to the patients and improves patient adherence to treatment when multiple interventions are required (Iwu & Holzemer 2014; Siapka et al. 2014; Tran et al. 2015).

Despite the benefits of the integration of HIV and AIDS services into PHC, a lack of human resources in many resource-limited settings, especially in rural areas, is still a challenge, which often hinders integration of services. Various strategies such as task-shifting were, therefore, introduced in countries with severe human resources shortages. Task-shifting involves redistributing of selected tasks from physicians to adequately trained nurses and from nurses to adequately trained lower-level health workers or lay providers (Fronczak et al. 2015).

In South Africa, the public ART programme was hospital-based. Patients were to be referred from PHC facilities to accredited hospitals for ART programmes and this caused patients to wait a long time; some even died before they could be initiated on ART. In an effort to reduce the burden placed on the hospital systems, many ART programmes were integrated into PHC facilities, the public health policy switched from doctor-based, hospital-centric ART services to decentralised provision of nurse-initiated management of antiviral therapy (NIMART). Recently, antiretrovirals (ARVs) are initiated daily in the PHC facilities; all patients follow a uniform patient flow, and patient load is evenly spread out among the existing staff. These resulted in a dramatic expansion in the number of people initiating ARTs, with approximately half of individuals in need of ART receiving treatment in 2011. According to Statistics South Africa (2011), access to ART has changed historical patterns of mortality and the number of AIDS related deaths declined consistently since 2007 from 345 185 in 2006 to 126 755 AIDS related deaths in 2017.

Best practices in the integration of HIV and AIDS services into PHC include integration of provider-initiated testing and counselling (PICT) into clinical services; integration of HIV services into antenatal, labour and delivery, postpartum and newborn care; TB and HIV care and treatment (TB-HIV co-management); integration of family planning into maternal and HIV care; and integration of sexually transmitted infection (STI) screening and management into chronic and acute care. Therefore, service integration includes integration of TB-HIV co-management, integration of HIV services into antenatal, labour and delivery, postpartum and newborn care; PMTCT; integration of family planning and sexual and reproductive health into HIV services and maternal care; integration of STI management into acute and chronic HIV care; and integration of HIV services into services for adolescents (UNAIDS 2013).

### Problem statement

The National Department of Health in South Africa, in response to the accelerated roll-out of ART, adopted the strategy of integrating HIV and AIDS services into PHC. The strategy was anticipated to decrease the burden of providing HIV services at existing facilities while making access to care for PLWHIV easier and ensuring improved health outcomes (Odeny et al. 2013). However, the shortage of human and material resources as an ongoing problem in South Africa, impinging the effective integration of HIV services into PHC. According to the integrated primary healthcare (IPHC) model, the process of integration requires functional health facilities with adequate infrastructure, equipment and human resources capable of providing services such as ART and PMTCT (Sibiya & Gwele 2013). There was insufficient evidence of whether the integration of HIV and AIDS services into PHC is effectively implemented, particularly in Vhembe district where the study was focused. The interest of the researcher emanated from the above deliberation. The researcher, therefore, explored the challenges of PHC nurses regarding the integration of HIV and AIDS services into PHC, as nurses are the ones responsible for the integration of HIV and AIDS services programmes at clinics and health centres.

### Aim

The overall aim of this study was to explore the challenges of PHC nurses regarding the integration of HIV and AIDS services into PHC in Vhembe district of Limpopo province, South Africa.

## Research method and design

### Context

The study was conducted at two selected health centres and 10 clinics located in Vhembe district of Limpopo province. A clinic is a facility at and from which a range of PHC services is provided and that is normally open eight or more hours a day based on the need of the community to be served while a health centre is a facility that normally provides PHC services, 24 h maternity, accident and emergency services and beds where healthcare users can be observed for a maximum of 48 h and which normally has a procedure room but not an operating theatre (Department of Health 2001)

Limpopo province has five districts and Vhembe is one of these districts. Vhembe district is located in the northern part of Limpopo province and it is bordered in the east by the Kruger National Park, South East Mopani District, South West Capricorn District, North East Botswana and North Zimbabwe. It is consisted of four local municipalities, namely Musina, Mutale, Thulamela and Makhado.

### Study design and population

A qualitative, explorative, descriptive and contextual design was used to conduct a study among professional nurses from clinics and health centres in Vhembe district of Limpopo province, South Africa. Qualitative research was selected to understand the challenges that nurses are experiencing while integrating HIV and AIDS services into PHC. Qualitative research is concerned with understanding the meaning of social interaction by those involved (Grove, Gray & Burns 2011). The study population comprised 10 PHC nurses from the clinics and 2 PHC nurses from health centres. Non-probability purposive sampling was used to select PHC nurses with more than 2 years of experience at clinics and health centres where HIV and AIDS services integration is practised.

### Data collection

The researcher as a student of the degree of doctor of philosophy, visited clinics and health centres with formal letters to request permission to conduct the study. Nurses were told about the nature and objectives of the study. Appointments were made with nurses who agreed to participate in the study. The researcher used an interview guide to facilitate the communication (one-on-one semi-structured interview) with participants. Participants were encouraged to talk freely about the challenges regarding the integration of HIV and AIDS services into PHC and each interview lasted for 45 min – 60 min. The researcher made observations and field notes were taken. Before commencing with the semi-structured interview, permission was sought to have a private room and all interviews were conducted at clinics or health centres. Twelve professional nurses were interviewed and the number of participants was determined by data saturation. The following central question was asked:

#### What are your challenges regarding the integration of HIV and AIDS services into primary healthcare?

To ensure that all responses from the participants were recorded, a digital voice recorder was used to record the responses of the participants. Participants were made aware that they were being recorded. Communication techniques such as probing, paraphrasing, summarising, clarification and listening were used to get in-depth information.

### Data analysis

Data analysis was performed concurrently with data collection and eight Tesch steps of open coding were followed (Creswell 2012). Coding was done by the researcher and the independent coder and after the coding of themes, they reached consensus regarding the final themes and sub-themes (see Table 1).

**TABLE 1:** Themes and sub-themes.

Theme	Sub-themes
Theme 1: Nurses have challenges related to healthcare recipients	1.1 Refusal of HIV testing 1.2 Non-adherence to the scheduled appointments 1.3 Non-acceptance of HIV status 1.4 Disclosure of HIV+ status problematic leading to lack of ART adherence
Theme 2: Challenges related to service delivery	2.1 High workload related to service integration 2.2 Insufficient consulting rooms 2.3 Inadequate number of staff members

HIV, human immunodeficiency virus; ART, antiretroviral therapy.

A literature control was conducted after data analysis to identify similarities and the uniqueness of the research. The purpose of the literature review was to put the findings in the context of what is already known. It also enabled the researcher to identify the relationships and variation between the present and previous studies as well as the potential contributions of the current study towards the knowledge pool that existed (Grove, Burns & Gray 2014:116; Polit & Beck 2012:133).

### Measures to ensure trustworthiness

Measures to ensure trustworthiness was ensured by using criteria of credibility which was ensured by prolonged engagement, persistent observation, triangulation, external checks such as peer debriefing and member check, and the researcher’s credibility. Purposive sampling of clinic and health centre managers was used to increase transferability, audio tape was transcribed by the researcher and cross-checked with the field notes taken by the researcher to ensure dependability and conformability was enhanced by the availability of raw data on audio-recorder and transcriptions to verify the themes.

### Research participants

The sample comprised professional nurses who were managing clinics or healthcare centres (Operational managers) in Vhembe district of Limpopo province, South Africa. Ten participants were from clinics while two participants were from healthcare centres. Data saturation occurred after interviewing 12 participants, who were three male professional nurses and nine female professional nurses. Their ages ranged between 45 and 55 years, with the working experience of more than 2 years at PHC facilities.

### Ethical considerations

Permission to conduct the study was obtained from the School of Health Sciences Higher Degree Committee, the University of Venda Ethics Committee (SHS/15/PDC/33/0502), the Limpopo Department of Health Provincial Research Committee and Vhembe district Department of Health as well as sampled health centres and clinics of Vhembe district. Informed consent was obtained from all nurses who participated in the study. The ethical principle of beneficence, respect for human dignity and justice was adhered to in the study.

## Results

Two main themes emerged from data analysis: nurses have challenges related to healthcare recipients, which included refusal of HIV testing, non-adherence to scheduled appointment, non-acceptance of HIV status and non-disclosure of HIV+ status, as well as challenges related to service delivery, which included high workload, insufficient consulting rooms and an inadequate number of staff.

### Theme 1: Challenges related to healthcare recipients

#### Refusal of HIV testing

Nurses at clinics and health centres are experiencing challenges of patients who refuse to be tested for HIV during consultation and this hinders HIV and AIDS services integration into PHC. Some patients even if they are tested do not come back for test results. Nurses also explained that pregnant women who do not accept their pregnancies often refuse HIV counselling and testing, as they are anxious because of unwanted pregnancy. Integration of HIV and AIDS services into PHC cannot be effective without PICT. According to the HIV testing service policy (2016), PICT should be offered to all persons attending clinical services in both the public and private sector regardless of whether they show signs or symptoms of HIV infection. This allows the healthcare provider to make medical decisions that would not be possible without knowledge of the patient’s HIV status:

‘Some patients refuse to be tested for HIV during their consultation and this is a challenge as we are expected to counsel and test all clients for HIV if we are to integrate HIV/AIDS services into other PHC programmes. With some clients; even if they are tested they don’t come back and when you do follow-up by trying to phone their phones are switched off and that is a challenge.’ (P1, female, 40 years old)

Another participant said:

‘We have a serious challenge with pregnant women with unwanted pregnancies, when a nurse counsels them for HIV they do not want to be tested as they are angry with their pregnancies, especially the teenagers.’ (P8, female, 50 years old)

Studies revealed reasons for refusal of HIV testing as lack of knowledge about a partner’s HIV status and not knowing anyone with HIV and AIDS, stigma attached to HIV, fear of break-up of relationships and lack of knowledge of the partner’s HIV status. Fears of stigma and discrimination from male partners were found to be more important negative influences on HIV test acceptance than fears of stigma and discrimination from others, such as friends, family, co-workers, health workers and others in the community (Mthethwa 2014; Onono et al. 2015; Setse & Maxwell 2014). These studies further revealed that fear of confidentiality violations was also a reason for refusing HIV testing and many people declined testing because they had tested recently, often in the same healthcare facility, and individuals did not perceive themselves to be at risk for HIV infection, usually because they were in long-term monogamous relationships and had been tested prior to or during these relationships.

#### Non-adherence to scheduled appointments by patients

Nurses from the clinics and health centres explained that they have a challenge when integrating HIV and AIDS services into PHC because of patients who do not adhere to their scheduled appointments. Non-adherence to the scheduled appointments hinders the integration of HIV and AIDS services into PHC; particularly in the case where polymerase chain reaction (PCR) for babies who are born of HIV-positive mothers are to be repeated. A participant responded in this manner:

‘We have a challenge when clients do not adhere to the scheduled appointment; particularly to those who have already started with ARVs when they become better they stop treatment saying that they are cured, okay.’ (P4, female, 42 years old)

‘We are experiencing problems with some of the mothers after delivery; when we are supposed to repeat PCR, they don’t turn-up; some would decide to stop Nevirapine for the baby then when we repeat PCR, we find that it is positive and that is a challenge.’ (P2, male, 45 years old)

The study conducted by Bigna et al. (2014) revealed that the most significant determinant of not attending medical appointments was the family member’s lack of formal education, which led to not understanding the importance of follow-up appointments. The study also revealed that the longer the time to the next appointment, the higher the failure rate of keeping appointments, which could be related to forgetfulness.

#### Non-acceptance of HIV status

Nurses at clinics and health centres have challenges when they come across patients who do not accept their HIV positive status. They explained that some patients become frustrated after they are told of their HIV positive status and that interferes with the whole process of HIV and AIDS service integration. A participant indicated that integrating HIV and AIDS services into PHC is a process, which must end with the initiation of ARVs and such initiation is not possible if the patient is not accepting of their HIV status:

‘Some patients become frustrated when they are informed about their HIV positive status in such a way that they would not like to frequently come to the clinic and that is challenging to the whole process of HIV and AIDS service integration. Integration of HIV services into PHC must continue with the initiation of ARVs for those who are eligible; so if the patient is not accepting the status, it becomes a challenge.’ (P5, male, 45 years old)

### Non-disclosure of HIV status

Nurses specified that patients do not want to disclose their HIV status to their relatives fearing that they would be rejected by their family members. This fact hindered service integration as there would be non-adherence to ARVs because patients would not want to be seen taking ARVs and integration is a continuous process, which must end with the patient improving by adhering to ARVs:

‘Patients aged 16–19 years would not want their HIV positive status to be known; mostly they do not have sufficient knowledge about HIV, they just take HIV as a dreadful disease. Firstly, if a girl is pregnant, she is afraid of her boyfriend and parents thinking that she will be rejected. Because of this fear of rejection as well as the unwanted pregnancy and HIV infection, she cannot disclose the status to them. This is a challenge because if the patient does not disclose the status, it means that there would be no one to support the patient with the treatment, which leads to non-adherence of ARV and the patient would not improve.’ (P10 female, 50 years old)

The findings of the current study support the study by Musumari et al. (2014), who argued that patients often do not disclose their HIV status out of fear of rejection and gossip and this causes a potential barrier to ART adherence.

### Theme 2: Challenges related to service delivery

#### High workload related to service integration

Nurses explained that integration of HIV services into PHC increases workload which has a negative impact on service delivery; nurses indicated that integrating HIV and AIDS services is time-consuming as they spend too much time with one patient, especially for the newly HIV diagnosed patients. Before integration, doctors initiated ARVs; however, since the integration has started, ARV initiation is done by nurses:

‘The workload is high; if the government or the department can be able to increase the number of personnel; it would be very, very simple for us to integrate HIV service into PHC as well to render quality care to our clients.’ (P3, female, 43 years old)

Another participant has said:

‘Workload is too much; ya, workload is too much. At first patients used to be initiated by doctors; but now we do initiate ourselves; it is a nurse’s programme, is no longer a doctor’s. You just consult a doctor if you encounter problems but you have to initiate. We initiate our own patients; sometimes if you refer the patient, they will just send the patient back.’ (P1, female, 40 years old)

According to Uebel et al. (2013), high workload with a large number of patients had complex effects on integration while the smaller size and staff complement of some clinics appeared to promote an integration of care. These authors further concluded that as long as nurses are expected to manage large number of patients each day in primary care, HIV and AIDS services are unlikely to be successfully integrated into PHC; more especially in countries like South Africa where criteria for ART eligibility have been widened and a large number of people are eligible for ART.

#### Insufficient consulting rooms

Nurses explained that they have insufficient consulting rooms to such an extent that in many clinics, post natal wards are used for HIV counselling and testing and has a negative impact on the integration of HIV and AIDS services into PHC services.

Participants have said:

‘We are running short of cubicles, we often use postnatal room when there is no patient at post natal, we use it for counselling and testing and that interferes with confidentiality when doing HIV testing and counselling. Sometimes patients leave the without being counselled for HIV because of lack of space, hence no integration and that is a challenge.’ (P9, female, 48 years old)

According to Crowley and Stellenberg (2014), essential HIV care and treatment services, as well as managerial and infrastructural support, are key components for ensuring quality services. These include the availability of sufficient consulting rooms.

#### Inadequate number of staff members

An adequate number of staff are needed for successful integration of HIV and AIDS services into PHC. However, nurses explained that there are an inadequate number of staff at clinics and health centres in Vhembe district and this impedes the integration of HIV and AIDS services into PHC. According to the IPHC model, the availability of human resources is mandatory to create an enabling environment for the HIV and AIDS service integration into PHC (Sibiya & Gwele 2013).

‘We are short staffed; you can find two clinical nurse practitioners consulting patients who are ±100 per day. And according to the guideline and other policies that I have seen; in order to give a proper service to a client in a day we are supposed to consult ± 30 patients per day. If the government or the department can be able to increase the number of staff; it would be very simple for us to integrate HIV/AIDS services in all programmes at the clinic.’ (P6, female, 48 years old)

Shortage of staff is related to difficulties in providing quality services such as full examination, excluding opportunistic infections and getting proper history information. Insufficient staff is a barrier for the integration of HIV and AIDS services into PHC (Mathibe, Hendricks & Bergh 2015:^5^; Topp et al. 2010:354). According to Crowley and Stellenberg (2014), the integration of HIV and AIDS services into PHC requires functional health facilities with adequate infrastructure, equipment and human resources capable of providing services such as ART; PMTCT, TB; and Sexual and Reproductive Health; good medical storage systems and guidelines that ensure quality assurance mechanisms; and functional logistics and supply chain management systems capable of providing an uninterrupted supply of commodities.

High workload related to service integration was also revealed as a challenge for PHC nurses when integrating HIV and AIDS services into PHC. These findings concur with the study conducted by Mathibe et al. (2015), which revealed that high workload is caused by staff shortages and increased activities such as counselling for new and follow-up clients; examination; routine investigations; amount of forms to be completed; management of side-effects and complications and self-dispensing and issuing treatment from consulting rooms.

## Study limitations

The study was conducted only at the clinics and healthcare centres in Vhembe district of Limpopo province and cannot be generalised.

## Recommendations

Based on the findings highlighted above, the following recommendations are suggested by the researcher:

### Recommendations for nursing practice

- There must be a clear policy on human resources that will include strategies for dealing with the migration of health staff to developed countries as well as the retention strategies.
- Planning for adequate space for HIV and AIDS service integration into PHC should accommodate the reality of existing basic clinical services.
- Policies for HIV and AIDS services need to be reviewed to inform the structuring of an environment that enables the integration of HIV and AIDS services into PHC.

### Recommendations for nursing education and training

- In response to increased burden of the disease and a growing population, the training of sufficient number of nurses with appropriate skills must be a human resource priority.
- There should be an expanded role of schools, colleges and university programmes of nursing education in developing continuing professional education in IPHC.

### Recommendations for nursing research

This study revealed that the clinic/health centre environments are not enabling to integrate HIV and AIDS services into PHC; the researcher, therefore, recommends that future research be conducted on the impact and feasibility of HIV and AIDS services integration into PHC.

## Conclusion and recommendation

Despite the benefits of integrating HIV services into PHC, the study revealed that PHC nurses face certain challenges that need attention for the effective integration of HIV and AIDS services into PHC. It is, therefore, recommended that clear policies on the integration of HIV and AIDS services into PHC be available. These policies should include rescheduling of appointments to give clients an opportunity to think about an HIV+ test as well as revising the human resource policy to reduce workload. HIV awareness campaigns should be done regularly to empower the communities with knowledge on issues related to HIV and AIDS.
